# Fatigue Performance of 3D-Printed Poly-Lactic-Acid Bone Scaffolds with Triply Periodic Minimal Surface and Voronoi Pore Structures

**DOI:** 10.3390/polym16152145

**Published:** 2024-07-28

**Authors:** Hamed Bakhtiari, Alireza Nouri, Majid Tolouei-Rad

**Affiliations:** 1 Centre for Advanced Materials and Manufacturing (CAMM), School of Engineering, Edith Cowan University, Joondalup, WA 6027, Australia; h.bakhtiari@ecu.edu.au; 2 School of Engineering, RMIT University, Melbourne, VIC 3001, Australia; alireza.nouri@rmit.edu.au

**Keywords:** bone scaffold, fatigue, compression, PLA, additive manufacturing

## Abstract

Bone scaffolds serve a crucial role in tissue engineering, particularly in facilitating bone regeneration where natural repair is insufficient. Despite advancements in the fabrication of polymeric bone scaffolds, the challenge remains to optimize their mechanical resilience. Specifically, research on the fatigue behaviour of polymeric bone scaffolds is scarce. This study investigates the influence of pore architecture on the mechanical performance of poly-lactic-acid (PLA) scaffolds under quasi-static and cyclic compression. PLA scaffolds with a 60% porosity were fabricated using extrusion-based 3D printing in various designs: Gyroid, Lidinoid, Fischer–Koch, IWP, and Voronoi. Results demonstrated that Gyroid scaffolds had the highest compressive strength (6.6 MPa), followed by Lidinoid, Fischer–Koch, IWP, and Voronoi designs. Increased strut thickness was linked to higher compressive strength. However, normalized fatigue resistance showed a different pattern. While scaffolds resisted fatigue cycles at low strain amplitudes, fatigue damage was observed at higher strains. Voronoi structures exhibited the highest normalized fatigue performance, enduring around 58,000 cycles at 85% strain amplitude, followed by Gyroid, Fischer–Koch, Lidinoid, and IWP structures. Enhanced fatigue performance in different topologies correlated with the minimum cross-sectional area of scaffolds. Given the importance of both static and fatigue strength, the Gyroid topology emerges as the superior choice overall.

## 1. Introduction

Bone fractures and defects can result from various factors, including trauma, osteoporosis, overuse, medical conditions, and nutritional deficiencies [1,2]. In 2000, an estimated 9 million bone fractures worldwide were solely due to osteoporosis [1]. The incidence of bone fractures has been found to increase considerably over the years, with 178 million new cases reported around the globe in 2019 [3], underlining the increased burden of bone injuries on global healthcare systems. To address this challenge, effective bone fracture repair techniques have been explored over the past two decades [4]. Conventional techniques such as autograft and allograft often encounter issues such as scarcity, infection risk, and immune rejection [5]. To overcome these challenges, bone tissue engineering (BTE) was developed, beginning in the late 20th century with the use of biodegradable polymeric scaffolds for cell transplantation [6]. The main cornerstones of BTE involve developing biocompatible scaffolds that support cell attachment and bone ingrowth, and which withstand external mechanical loads. These engineered scaffolds aim to replicate the bone’s extracellular matrix by providing a three-dimensional (3D) structure that promotes cell growth, differentiation, and vascularization. An ideal scaffold should be biocompatible, biodegradable, and have proper mechanical properties [7].

Recent advancements in 3D printing technology have enabled the development of customized bone tissue scaffolds featuring different materials and complex designs. 3D printing techniques used for bone tissue engineering can be categorized into beam-based 3D printing techniques such as SLS [8] and SLM [9] and beamless techniques such as material jetting [10], binder jetting [11], and FDM [12]. Beam-based techniques (SLS, SLM) offer high precision and superior mechanical properties but are limited by high costs, material restrictions, and low cell viability. Beamless techniques (material jetting, binder jetting, FDM) provide greater material versatility, lower costs, and high cell density but may compromise on resolution and mechanical strength [13].

**Figure 1:**
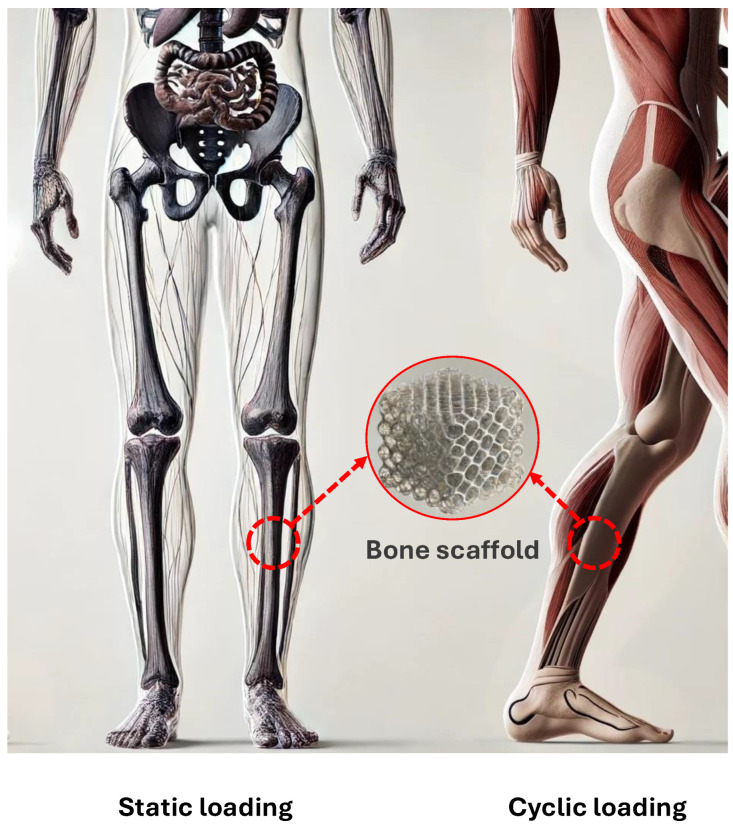
Static and cyclic loading during daily human activities (created by AI).

Bone scaffolds experience various physiological forces, such as tension, compression, and bending, under both static and cyclic conditions throughout their service life. Figure 1 illustrates the static and cyclic loading experienced by bone scaffolds during standing and walking. Loading conditions significantly impact the durability of bone scaffolds. Daily activities like walking, running, cycling, or climbing induce repetitive stresses that can lead to bone stress injury and fatigue failure [14]. Thus, an ideal bone scaffold should withstand both static and fatigue stresses throughout the bone healing period. Despite extensive research on the static performance of polymeric bone scaffolds, their fatigue behaviour remains relatively unexplored. Bakhtiari et al. [15] provided a detailed review of the factors influencing the fatigue resistance of bone scaffolds, highlighting material characteristics, in vivo conditions, topological features, and loading conditions as the most influential parameters. From a material perspective, the fatigue resistance of polymeric bone scaffolds can be improved by using higher-molecular-weight polymers or by reinforcing them with materials such as bioceramics or metals. Conversely, being exposed to body fluids can cause polymer degradation through hydrolysis and oxidation and consequently lead to a reduction in mechanical and fatigue resistance of bone scaffolds. On the other hand, the gradual formation of bone tissue within scaffold pores enhances mechanical support and improves the overall fatigue resistance of bone scaffold [15].

**Figure 2:**
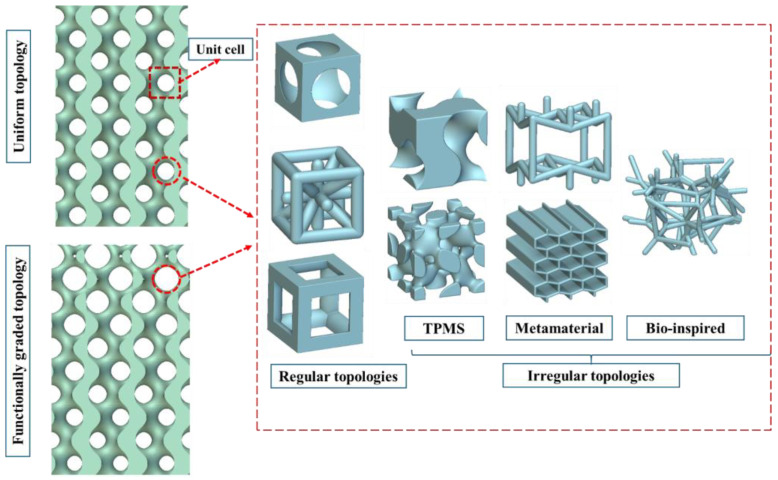
Illustration of different topologies used in designing bone scaffolds.

Topological features such as porosity, pore size, shape, and distribution of pores within a scaffold significantly influence its mechanical and fatigue characteristics [16]. While higher porosity can enhance bone ingrowth [17], it also compromises compressive strength and fatigue resistance of the polymeric scaffold [18,19]. Similar to porosity, size of the pores influences both biological and mechanical characteristics of bone scaffolds [20]. Although pore sizes larger than 300 µm are favourable for bone ingrowth [21], they often reduce the mechanical strength of scaffolds. Research has demonstrated that large pores in scaffolds can expedite crack initiation and propagation, leading to premature fatigue failure. In contrast, smaller pore sizes can significantly improve the scaffold’s resistance to fatigue crack propagation [22]. Therefore, a trade-off must be managed between porosity and pore size in scaffold design to optimize both bone ingrowth and mechanical strength [21]. The shape of pores also plays a crucial role in the mechanical behaviour of scaffolds. Pore shapes in scaffolds can vary widely, including both regular geometries (such as circle, triangle, cube [23], tetrahedron and hexagon [24]) and irregular shapes (triply periodic minimal surface (TPMS) [25], metamaterials [26], bioinspired [27], and functionally graded topologies [28,29]). Figure 2 illustrates the variation in pore size, shape, and distribution within the scaffold. The image highlights differences in pore morphology, including both round and irregular shapes, as well as variations in pore size and nonuniform pore distribution across the scaffold.

Few studies have explored the influence of pore architecture on the fatigue behaviour of polymeric bone scaffolds. Orthogonal (0/90°) pores exhibited higher compressive strength [30,31,32] and superior fatigue performance [18,19] compared to isometric patterns, likely due to increased slippage resistance between layers. In another study, Liang et al. [24] demonstrated the low cycle fatigue behaviour of 3D-printed PLA scaffolds with hexagonal, tetragonal, and wheel-like topologies, all having similar porosities. The results indicated the superiority of tetragonal design under cyclic loading, enduring 4400 fatigue cycles, attributed to its increased number of junction points, which provided enhanced mechanical support. Gong et al. [33] investigated 3D-printed PLA scaffolds with different pore architectures under compression and demonstrated that triangular pores possessed higher strength than circular topology. However, circular pores performed better under dynamic loading.

Complex pore architectures such as TPMS, Voronoi tessellation, functionally graded structures, and auxetic metamaterials in polymers have demonstrated promising load-bearing capacities [34,35,36,37,38,39,40,41]. Notably, the TPMS structure has shown potential in reducing stress concentration due to their smooth surface transitions at strut junctions [42]. TPMS structures feature continuous and smooth surfaces that maintain zero mean curvature at all points of their surfaces. This geometric characteristic is advantageous for higher mechanical and fatigue performance because it facilitates uniform stress distribution across the structure. The absence of sharp angles and discontinuities, which can concentrate stress and initiate cracks, enhances the structural durability under both static and dynamic loads. Voronoi tessellation has also gained attention in the biomedical field for its ability to mimic the natural, irregular patterns of bone microarchitecture. This geometry exhibits load-bearing and permeability characteristics that make it effective for bone scaffold applications [28,43]. However, the fatigue performance of these structures in polymeric bone scaffolds remains poorly understood, as it is underrepresented in the current literature. Without a comprehensive knowledge of how these scaffolds respond to cyclic loading, there is a risk of premature failure, which can compromise the structural integrity and longevity of the scaffold. To address this issue, the present study investigates the fatigue performance of PLA bone scaffolds with various pore architectures under compressive loading at different strain amplitudes. PLA scaffolds with Gyroid, Lidinoid, Fischer–Koch, Voronoi, and IWP pore structures were 3D-printed using an extrusion-based technique, and their static and fatigue strengths under compression were examined. This study underscores the importance of selecting appropriate pore architectures to balance the mechanical strength and fatigue resistance of PLA bone scaffolds, thereby providing valuable insights for optimizing scaffold designs in bone tissue engineering applications.

## 2. Materials and Methods

### 2.1. Bone Scaffold Fabrication

PLA was selected as the base material for bone scaffolds. Scaffold samples, featuring Gyroid, Lidinoid, Fischer–Koch, Voronoi, and IWP pore structures, were designed using nTopology 4.11.2 software. To comply with the standard test procedures for strength measurements (ASTM D1621-16 [44]), samples were designed as rectangular prisms with dimensions of 12.7 × 12.7 × 25.4 mm. Table 1 provides schematics of the topologies used in this study, along with the formulations employed to generate each topology. α, β, and γ denote constants related to the unit cell size (L) in x, y, and z, respectively; c is the offset parameter, which equals zero for a single unit cell of the TPMS.

To isolate the effect of pore shape, the porosity and unit cell size of all scaffolds were maintained at 60% and 2.5 mm, respectively, creating a 10 × 5 pore network within the scaffold’s structure. Unit cell sizes ranging from 1 to 2.5 mm and porosities between 50% and 85% are considered optimal for biomedical implants [45]. Raise3D premium PLA filament (Raise 3D Technologies Inc., Irvine, CA, USA) with a diameter of 1.75 mm was used as the feedstock for the 3D printer. Fused deposition modelling (FDM) was employed to produce the scaffolds using a Raise3D pro2 Plus 3D printer (Raise 3D Technologies Inc., Irvine, CA, USA). The selected printing parameters included a layer thickness of 0.05 mm, an extrusion width of 0.65 mm, a nozzle temperature of 220 °C, and a print speed of 45 mm/s. These parameters were determined based on the findings of a parametric study conducted in our prior research [12,46]. Table 2 provides the physical and mechanical characteristics of the PLA filament used in this study.

**Figure 3:**
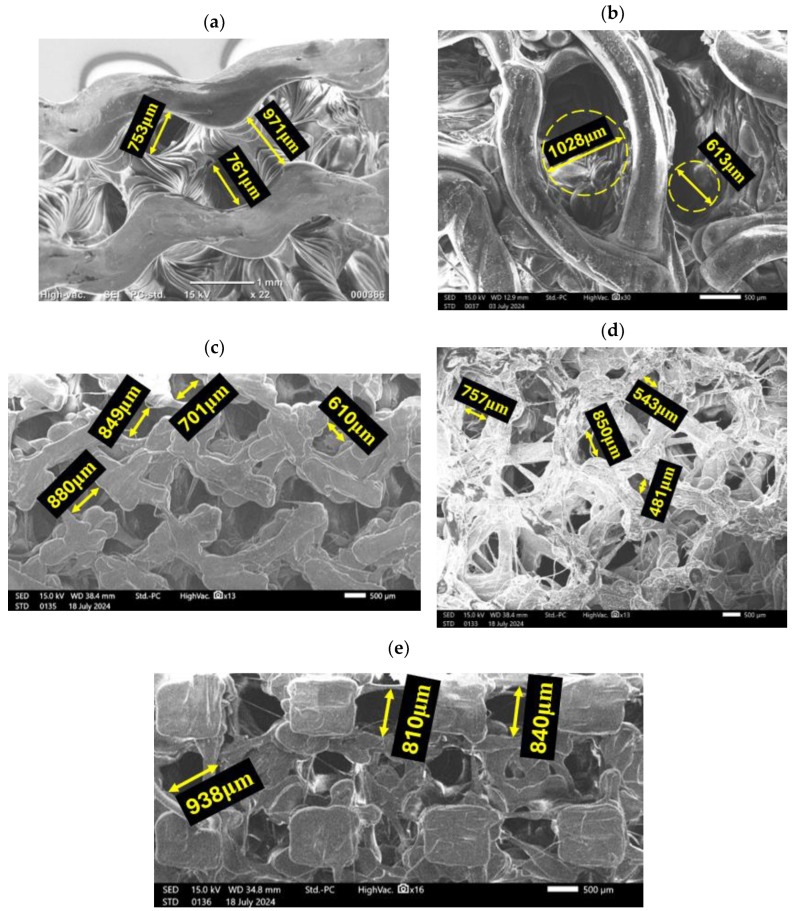
SEM images of 3D-printed (**a**) Gyroid, (**b**) Lidinoid, (**c**) Fischer–Koch, (**d**) Voronoi, and (**e**) IWP scaffolds.

Table 3 summarizes the initial design and 3D-printed bone scaffolds, along with the topological characteristics of each design. SEM images of 3D-printed scaffolds are also provided in Figure 3. As shown in the table, although the porosity of all scaffolds is identical (i.e., 60%), the pore sizes vary between 700 and 900 µm due to the differences in pore shapes across various topologies. This range aligns with biological standards for scaffolds, which suggest that high porosity and interconnected channels with pore diameters ranging from 300 to 900 μm are optimal for bone tissue engineering [48].

### 2.2. Quasi-Static Compression Test

Quasi-static compression tests were conducted on scaffold specimens according to ASTM D1621-16 [44], which outlines methods for assessing the compressive properties of rigid cellular plastics. An Instron 8801 machine equipped with a double-acting servo hydraulic actuator was utilized to apply compressive loads. The length, width, and height of the specimens were measured at three points each and rounded to the nearest 0.01 mm. The average of these dimensions was used to calculate the cross-sectional area and volume of the scaffolds. Specimens were subjected to uniaxial loading at a speed of 1.3 mm/min until complete failure. Load-deformation data were recorded at a frequency of 20 Hz, and stress–strain data were derived by dividing the load by the cross-sectional area and the deformation by the specimen’s length.

**Figure 4:**
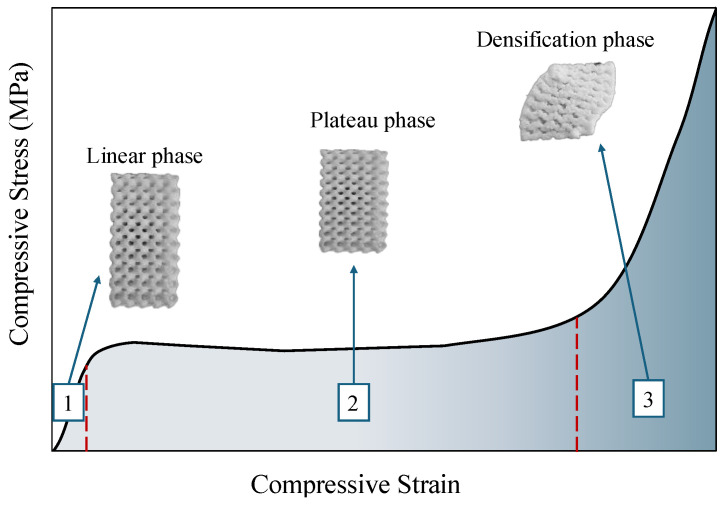
Typical compression behaviour of cellular solids, such as foams and porous scaffolds, is depicted in the stress–strain curve, which shows three phases: linear elastic (reversible deformation with linear stress increase), plateau (constant stress as cell walls collapse), and densification (steep stress rise as pores collapse and material density increases).

Figure 4 depicts the typical compression behaviour observed in cellular solids such as foams and lattice structures. The linear elastic region is characterised by the linear, reversible deformation of the scaffold under compressive force. Compressive strength and yield strain of the scaffold are determined by continuing the compression until the deformation results in permanent changes to the scaffold. This point is identified as the intersection of the stress–strain curve and a line parallel to the linear region, originating from a 0.2% strain offset [49,50]. After reaching the yield point, the cell walls begin to buckle or collapse, leading to the plateau region. This region is characterised by relatively constant stress because the collapsing pore structure dissipates the applied energy without significantly increasing the overall stress. Further compression moves the scaffold into the densification region, where most pores have collapsed, causing a sharp increase in material density. In this stage, the remaining solid framework bears the load, resulting in a steep rise in stress with additional strain [16,51]. Compressive modulus, compressive strength, plateau stress, and densification strain for each scaffold were derived from the stress–strain graphs. The compressive modulus was calculated by drawing a tangent to the linear region of the stress–strain curve and determining its slope using Equation (1):

(1) $$E=\frac{F\times h_{0}}{A_{0}\times ∆h}$$

where *E*, *F*, *h*_0_, *A*_0_, and ∆*h* denote the modulus of elasticity in compression, compressive load, initial specimen height, initial cross-sectional area, and deformation, respectively. Plateau stress was calculated where the stress–strain curve stabilizes. The densification strain was also measured at the point where the plateau line intersects with the tangent line drawn to the densification region.

### 2.3. Fatigue (Cyclic) Test

Strain-controlled fatigue tests at various strain levels (0.004ε, 0.015ε, 0.025ε, and 0.03ε) were conducted using an Instron 8801 device. Daily activities such as walking and uphill running can induce strains on bones from 0.0004ε to 0.002ε [15,52]. To maintain the loading within the elastic range, the yield strain for each sample was established via compression tests, ensuring that the applied fatigue loads did not exceed the elastic limit. Each scaffold was initially preloaded to approximately 0.2 MPa and then subjected to 100,000 sinusoidal compressive cycles at a frequency of 5 Hz. Force and deflection data were collected, and compressive stress, strain, and modulus were calculated for each cycle. Fatigue damage in polymeric scaffolds manifests as stiffness loss. According to various sources in the literature, fatigue failure in natural bone occurs when the initial stiffness is reduced by 50 to 90% [53,54,55]. In the present study, the slope of the linear region of the loading force–displacement curve was used to determine the elastic stiffness. The reduction in elastic stiffness is defined by Equation (2):

(2) $$\mathrm{Stiffness}\;\mathrm{reduction}(\%)=\frac{E_{0}-E_{cycle}}{E_{0}}\times 100$$

where *E*_0_ and *E_cycle_* denote the initial elastic stiffness and the elastic stiffness at the current cycle, respectively. The experiment was terminated when either a 60% reduction in the initial scaffold stiffness occurred or at the completion of 100,000 cycles, whichever came first.

## 3. Results and Discussion

### 3.1. Geometrical Features of Bone Scaffolds

**Figure 5:**
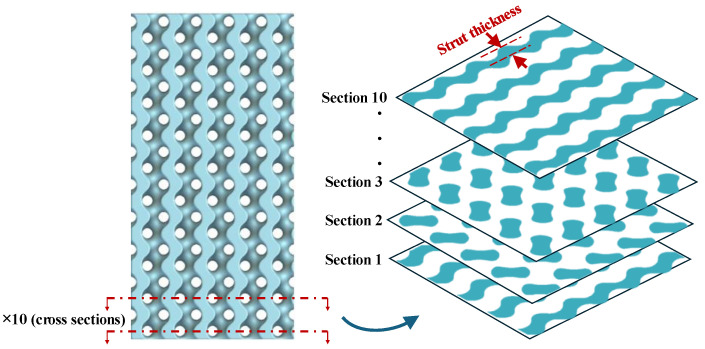
Measurement of strut thickness and cross-sectional area at ten different sections spanning the entire height of a repeating unit cell for each scaffold.

To correlate the mechanical properties of the scaffolds with their geometries, geometrical features such as strut thickness and cross-sectional area were derived from the designed CAD files. It is important to note that the porosity and pore size of all scaffolds fall within the same range. Strut thickness and cross-sectional area for each scaffold were measured at 10 different sections across the entire height of a repeating unit cell, as illustrated schematically in Figure 5.

Table 4 summarizes the cross-section, minimum cross-sectional area, and average strut thickness for various pore topology designs, including Gyroid, Lidinoid, Fischer–Koch, IWP, and Voronoi structures. Geometrical data provided in Table 4 indicate no specific relationship between strut thickness and minimum cross-sectional area; a higher strut thickness does not necessarily correlate with a larger cross-sectional area. For example, the minimum cross-sectional area of the Voronoi design is approximately 13.6% greater than that of the Lidinoid, yet its struts are, on average, 27.6% thinner. In fact, these two factors are independent and vary according to the topological design of the scaffolds.

### 3.2. Compressive Properties

**Figure 6:**
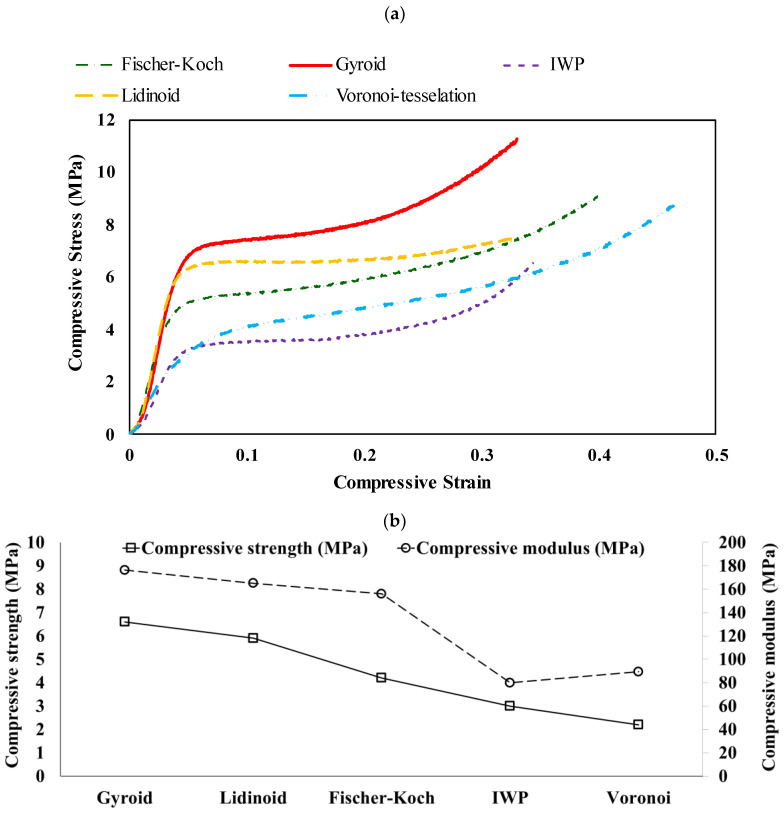
(**a**) Stress–strain curves and (**b**) compressive properties of PLA scaffolds, demonstrating the relationship between applied stress and the resulting strain, as well as the elastic modulus for various scaffold designs.

The stress–strain curves and compressive properties of PLA scaffolds are shown in Figure 6. Gyroid scaffolds exhibited the highest compressive properties, followed by Lidinoid, Fischer–Koch, IWP, and Voronoi topologies. As detailed in Table 5, the Gyroid scaffold demonstrated the highest compressive strength at 6.6 MPa, plateau stress at 7.7 MPa, a compressive modulus of approximately 176 MPa, and a densification strain of 0.26. In contrast, the Voronoi and IWP scaffolds exhibited the lowest compressive strength, around 2 to 3 MPa, and a compressive modulus of approximately 80 to 90 MPa, respectively. Unlike the other structures, the Voronoi scaffolds displayed no plateau region, indicating that densification begins immediately at the onset of plastic deformation.

**Figure 7:**
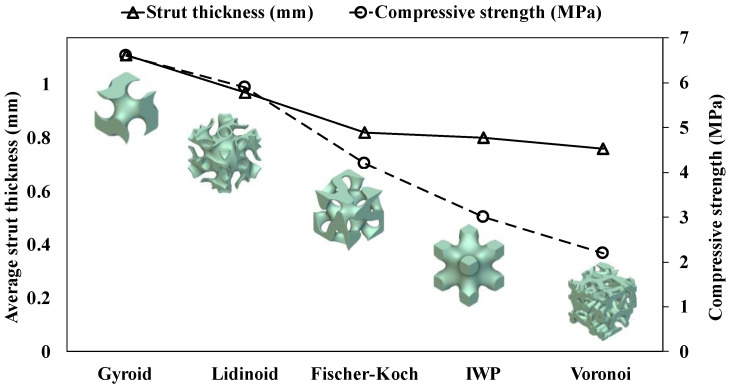
Change in average strut thickness across various topological scaffold structures.

The increase in compressive properties (i.e., compressive strength, compressive modulus, plateau stress, and densification strain) correlates with greater strut thickness, as depicted in Figure 7. The Gyroid scaffold exhibits the highest strut thickness, approximately 1.1 mm, followed by the Lidinoid, Fischer–Koch, IWP, and Voronoi scaffolds. The same trend can be seen in their compressive properties. This finding is consistent with the results obtained by Torres et al. [27], who reported that even a small increase in the thickness of rod-like struts (structural elements oriented perpendicular to the applied load) can significantly enhance the structural strength of a polymeric scaffold by 10 to 100 times while having minimal impact on density and stiffness.

### 3.3. Fatigue Properties

**Figure 8:**
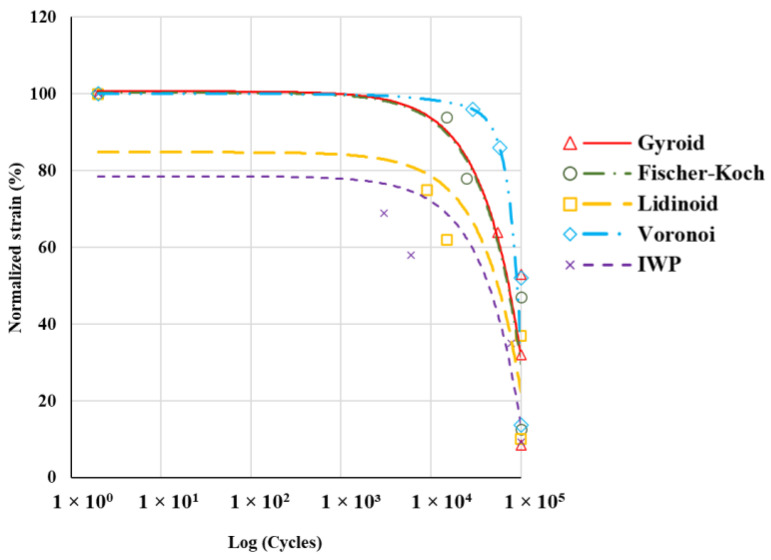
Normalised ε–N curves illustrate the fatigue performance of various topologies. The curves depict the relationship between normalised strain and the number of cycles to failure for each scaffold topology.

The fatigue performance of various topologies is shown in Figure 8 as normalised ε–N curves. Normalisation involves scaling the applied strain to the yield strain for each scaffold according to Equation (3):

(3) $$\mathrm{Normalised}\;\mathrm{strain}\left(\%\right)=\frac{\mathrm{Applied}\;\mathrm{strain}}{\mathrm{Yield}\;\mathrm{strain}}\times 100$$

In all topologies, increasing the strain amplitude led to a decrease in the number of cycles to failure. This is because higher strain amplitudes impose greater cyclic stress on the material, accelerating fatigue damage accumulation [12]. At low strain amplitudes (<15%), all scaffolds could endure fatigue loading. However, increasing the normalised strain to 35% resulted in the failure of the IWP topology at the 78,000th cycle. At this strain level, all other scaffolds withstood the fatigue cycles. Further increasing the normalised strain to the range of 65–70% caused the failure of all topologies. At this strain level, the IWP, Lidinoid, and Gyroid scaffolds failed at 3000, 15,000, and 55,000 cycles, respectively.

The Voronoi topology exhibited the highest normalised fatigue resistance, followed by the Gyroid, Fischer–Koch, and Lidinoid topologies. In contrast, the IWP topology showed the lowest normalised fatigue performance among the studied designs. These results suggest a significant difference between the fatigue performance of topologies and their behaviour under quasi-static loading. Despite having lower compressive properties, the Voronoi topology outperformed all other topologies in cyclic loading. The observed differences arise from the distinct failure mechanisms under static and dynamic loads [56]. Cubic structures generally show superior strength under static compressive forces [57], whereas structures with circular pores perform better in cyclic loading as a result of their more uniform stress distribution and reduced stress concentration [33]. In a study, Haddock et al. investigated the fatigue strength of vertebral and bovine tibial trabecular bones with varying porosities and found that they exhibited similar fatigue strength despite differences in yield strength [55]. Likewise, a study by Gong et al. on 3D-printed PLA scaffolds revealed that while triangular pore architectures had higher compressive strength, circular pore architectures provided better dynamic stability owing to a more even stress distribution [33].

In the present study, the analysis of the cross-sectional areas of various topologies revealed that the cross-sectional area perpendicular to the applied load plays a significant role in withstanding cyclic loading. These cross-sections provide mechanical support and effectively distribute the applied load across the structure. Since damage typically initiates at the weakest cross-sections, smaller cross-sections are more susceptible to damage under cyclic loading. Consequently, applying the same load to a smaller surface area leads to greater damage and a reduction in the overall stiffness of the structure.

**Figure 9:**
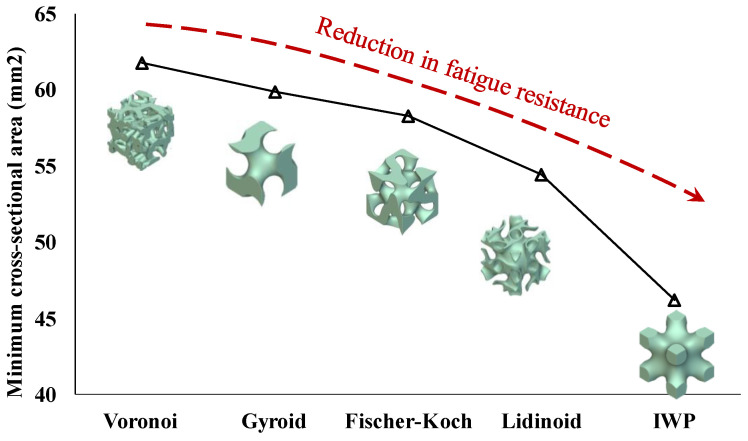
Minimum cross-sectional areas of various topological scaffold structures.

Figure 9 compares the minimum cross-sectional areas of the topologies examined. As shown, scaffolds with smaller cross-sectional areas tend to have lower fatigue resistance, while those with larger cross-sectional areas exhibit higher fatigue resistance. This is consistent with the findings reported in the literature [24], indicating that scaffolds with a higher number of junction points (resulting in a higher cross-sectional area) possess higher fatigue resistance.

## 4. Conclusions

PLA is an affordable, accessible, and 3D-printable material that has been approved by the FDA for biomedical use. However, its suitability as a material for biomedical implants is questioned due to its relatively low mechanical strength. While numerous studies have investigated the strength of PLA scaffolds under quasi-static conditions, research on their fatigue properties remains scarce. Given that scaffolds are porous structures, their pore architecture significantly impacts their fatigue durability when implanted in the body. The present study explored the impact of various pore architectures on the mechanical and fatigue performance of PLA bone scaffolds with 60% porosity. An extrusion-based 3D printing technique was used to fabricate five pore structures, including Gyroid, Lidinoid, Fischer–Koch, IWP, and Voronoi. The key conclusions drawn from this study are as follows:

- The Gyroid topology exhibited the highest compressive properties, including compressive strength (6.6 MPa) and modulus (176.3 MPa), making it the most robust structure under static loads.
- The Voronoi topology showed the lowest compressive strength (~2.2 MPa) and modulus (~89.3 MPa) but demonstrated superior normalised fatigue resistance, followed by Gyroid topology.
- Increased strut thickness correlated with higher compressive properties. The Gyroid structure, with the thickest struts, also had the highest compressive performance.
- Scaffolds with larger cross-sectional areas generally exhibited better fatigue resistance. The larger area provided more mechanical support and effective load distribution, reducing localized damage and enhancing longevity under cyclic loads.
- Given that both static and fatigue strength are critical for the efficacy of bone scaffolds, the Gyroid topology emerges as the superior choice overall.

While this study explored the fatigue performance of different topologies, future work can utilize the potential of structural optimization for enhancing the fatigue resistance of polymeric bone scaffolds. Since higher mechanical strength often compromises the biological performance of bone scaffolds due to reducing the pore size and porosity, multiobjective optimization of scaffold structures is necessary. While this paper demonstrates that strut thickness and cross-sectional area play key roles in the mechanical and fatigue strength of PLA bone scaffolds, stress concentration is expected to be among other influencing factors that can be investigated through numerical simulations in future works. With increasing interest in patient-specific and custom-designed polymeric bone scaffolds, these findings offer valuable insights for biomedical engineers and can help expand their application in clinical settings.

## Figures and Tables

**Table 1:** Schematic representations of the topological designs utilized in this study, accompanied by the mathematical formulations used to create each structure (*X*, *Y*, and *Z* denote the Cartesian coordinates in 3D space).

Topology	Formulation
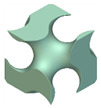 Gyroid	sin⁡Xcos⁡Y+sin⁡Ycos⁡Z+sin⁡Zcos⁡X
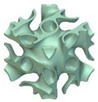 Lidinoid	$\sin\left(2X\right)\cos\left(Y\right)\sin\left(Z\right)+\sin\left(2Y\right)\cos\left(Z\right)\sin\left(X\right)+\sin\left(2Z\right)\cos\left(X\right)\sin\left(Y\right)-\cos\left(2X\right)\cos\left(2Y\right)-cos(2Y)\cos\left(2Z\right)-\cos\left(2Z\right)\cos\left(2X\right)$
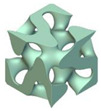 Fischer–Koch	$\cos\left(2x\right)\sin\left(y\right)\cos\left(z\right)+\cos\left(2y\right)\sin\left(z\right)\cos\left(x\right)+\cos\left(2z\right)\sin\left(x\right)\cos\left(y\right)$
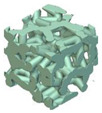 Voronoi	Randomly distributed set of points connected by irregularly shaped struts
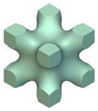 IWP	$2\left(\cos X\cos Y+\cos Y\cos Z+\cos Z\cos X\right)-\left(\cos2X+\cos2Y+\cos2Z\right)$

**Table 2:** Physical and mechanical characteristics of the PLA filament [47].

Physical and Mechanical Properties	Value
Density (g/cm^3^)	1.2
Glass transition temperature (°C)	62.3
Melting temperature (°C)	150.9
Young’s modulus (MPa)	2681 ± 215
Tensile strength (MPa)	40 ± 1
Elongation at break (%)	2.5 ± 0.6
Bending strength (MPa)	68 ± 2

**Table 3:** Details of the initial design and 3D-printed bone scaffolds, along with the topological characteristics of each design.

TPMS Structure	Designed Scaffold	3D-Printed Scaffold	Porosity (%)	Pore Size (µm)
Gyroid	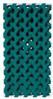	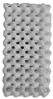	60	~748
Lidinoid	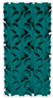	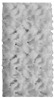	60	~883
Fischer–Koch	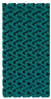	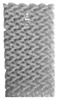	60	~682
Voronoi	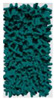	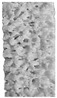	60	~708
IWP	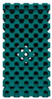	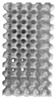	60	~936

**Table 4:** Geometric characteristics of various pore topologies.

Geometric Characteristics	Voronoi	IWP	Fischer–Koch	Lidinoid	Gyroid
Cross-section	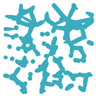	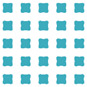	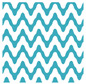	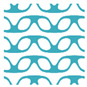	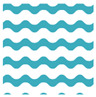
Minimum cross-sectional area (mm^2^)	61.8	46.2	58.3	54.4	59.9
Average strut thickness (mm)	0.76	0.80	0.82	0.97	1.1

**Table 5:** Compressive properties of PLA scaffolds.

TPMS Scaffold	Porosity (%)	Pore Size (μm)	Yield Strain	Compressive Strength (MPa)	Compressive Modulus (MPa)	Plateau Stress (MPa)	Densification Strain
Voronoi	60	710	0.029	2.2	89.3	−	0.05
IWP	60	936	0.043	3.0	79.8	3.6	0.19
Fischer–Koch	60	682	0.032	4.2	156	5.6	0.25
Lidinoid	60	883	0.040	5.9	165	6.6	0.23
Gyroid	60	750	0.047	6.6	176.3	7.7	0.26

## Data Availability

The raw data supporting the conclusions of this article will be made available by the authors on request.
